# Adverse childhood experiences and prevalence of type 2 diabetes in a nationwide study of women

**DOI:** 10.1093/eurpub/ckaf079

**Published:** 2025-06-10

**Authors:** Elísabet U Gísladóttir, Hilda B Daníelsdóttir, Huan Song, Marín D Bjarnardóttir, Arna Hauksdóttir, Arna Guðmundsdóttir, Diego Yacamán-Méndez, Edda B Thordardottir, Gunnar Tomasson, Harpa Rúnarsdóttir, Jóhanna Jakobsdóttir, Fang Fang, Unnur A Valdimarsdóttir, Thor Aspelund

**Affiliations:** Centre of Public Health Sciences, Faculty of Medicine, University of Iceland, Reykjavik, Iceland; Unit of Integrative Epidemiology, Institute of Environmental Medicine, Karolinska Institutet, Stockholm, Sweden; Centre of Public Health Sciences, Faculty of Medicine, University of Iceland, Reykjavik, Iceland; Unit of Integrative Epidemiology, Institute of Environmental Medicine, Karolinska Institutet, Stockholm, Sweden; Centre of Public Health Sciences, Faculty of Medicine, University of Iceland, Reykjavik, Iceland; West China Biomedical Big Data Center, West China Hospital, Sichuan University, Chengdu, China; Centre of Public Health Sciences, Faculty of Medicine, University of Iceland, Reykjavik, Iceland; Centre of Public Health Sciences, Faculty of Medicine, University of Iceland, Reykjavik, Iceland; Landspítali-The National University Hospital of Iceland, Reykjavik, Iceland; Department of Global Public Health, Karolinska Institutet, Stockholm, Sweden; Centre of Public Health Sciences, Faculty of Medicine, University of Iceland, Reykjavik, Iceland; Centre of Public Health Sciences, Faculty of Medicine, University of Iceland, Reykjavik, Iceland; Centre of Public Health Sciences, Faculty of Medicine, University of Iceland, Reykjavik, Iceland; Centre of Public Health Sciences, Faculty of Medicine, University of Iceland, Reykjavik, Iceland; Unit of Integrative Epidemiology, Institute of Environmental Medicine, Karolinska Institutet, Stockholm, Sweden; Centre of Public Health Sciences, Faculty of Medicine, University of Iceland, Reykjavik, Iceland; Unit of Integrative Epidemiology, Institute of Environmental Medicine, Karolinska Institutet, Stockholm, Sweden; Department of Epidemiology, Harvard TH Chan School of Public Health, Boston, MA, United States; Centre of Public Health Sciences, Faculty of Medicine, University of Iceland, Reykjavik, Iceland

## Abstract

We aimed to examine the association between adverse childhood experiences (ACEs) and type 2 diabetes in a nationwide cohort of Icelandic women, and to assess the mechanisms through which it is mediated. We used cross-sectional data from the nationwide-representative Stress-And-Gene-Analysis cohort, including 26 952 Icelandic women aged 18–69 years who self-reported exposure to 13 types of ACEs and adult diagnosis of type 2 diabetes. Modified Poisson regression was used to quantify the association between ACEs and type 2 diabetes, adjusting for age and childhood deprivation. We used causal mediation analysis to test whether adult body mass index (BMI), smoking, and socioeconomic factors mediated the association. Among a sample with a mean age of 44.2 (13.6), 780 (2.9%) women reported a diagnosis of type 2 diabetes. We observed a dose–response relationship between the number of ACEs and type 2 diabetes, of which women with five or more ACEs had almost double the prevalence compared to those with no ACEs (2.0% vs. 3.9%; prevalence ratio 1.90 [1.50–2.42]). Mediation analysis suggested adult BMI, smoking, and socioeconomic factors collectively explained 35.3% (16.5–53.6%) of the association but ACEs remained directly associated with type 2 diabetes (natural direct effect odds ratio 1.64 [1.25–2.26]). Bullying and sexual abuse were independently associated with a higher prevalence of type 2 diabetes. These findings suggest that adverse childhood experiences are associated with type 2 diabetes in adult women, partially mediated through adult BMI, smoking, and socioeconomic factors.

## Introduction

‘Adverse childhood experiences’ (ACEs) are alarmingly common, with studies indicating over half the population to have been exposed ACEs [1, 2]. The long-term health consequences of these experiences, which include abuse, neglect, or growing up in a dysfunctional home environment [3], are well documented. ACEs are associated with elevated risks of both psychiatric and somatic health outcomes in adulthood, including premature mortality [4–6]. Moreover, an accumulation of multiple ACEs further exacerbates the risk of adverse health outcomes [3, 7, 8].

While ACEs have repeatedly been found to be associated with type 2 diabetes [7, 9, 10], not all studies have confirmed such associations [8, 11, 12]. Discrepancies in findings include differences in sex-specific associations between ACEs and type 2 diabetes, with some studies indicating no association among women [9, 13, 14]. Furthermore, many studies consider only a limited number of ACEs, typically physical or sexual abuse, while the link between peer violence, such as bullying, and type 2 diabetes has not been studied previously. Mechanisms behind the association between ACEs and type 2 diabetes have not been extensively studied but existing research suggests socioeconomic, psychological, and lifestyle factors to play a role [15, 16]. Therefore, we leveraged the nationwide-representative Icelandic Stress-And-Gene-Analysis (SAGA) Cohort to comprehensively study the association between the cumulative burden as well as 13 distinct ACEs and type 2 diabetes among adult women, and to quantify the extent to which it is mediated by adult body mass index (BMI), smoking, and socioeconomic factors.

## Research design and methods

### Study sample and procedure

We used baseline data from the Icelandic SAGA Cohort, an ongoing nationwide-representative cohort study of lifetime stressors and women’s health. Between 2018 and 2019, all women aged 18–69 years living in Iceland were invited to participate in the study (≈104 197) by filling in an extensive online questionnaire regarding lifetime stressors (including ACEs), diseases (including type 2 diabetes), and current symptoms of physical and mental morbidities. A total of 30 403 women participated (approximately 30% of the target population), representing the Icelandic female population well in terms of age, education, income, and region of residence [17]. From the present study we excluded women with missing information on ACEs (*n* = 956 or 3.1%), or diabetes (*n* = 2381 or 7.8%). To ensure a reasonable chronology of the events, those diagnosed with type 2 diabetes before age 18 were excluded from the analysis (*n* = 4). We also excluded women with type 1 diabetes (*n* = 110), resulting in a final analytic sample of 26 952 women (Supplementary Fig. S1). To corroborate how the prevalence of self-reported type 2 diabetes in the present study compares to other reported estimates, we used age-specific prevalence from a previous nationwide study that used register data on prescriptions of diabetes medication [18].

This study was approved by the National Bioethics Committee of Iceland (IRB number 17-238).

### Adverse childhood experiences

ACEs were measured with a modified version of the Adverse Childhood Experiences International Questionnaire, developed by the World Health Organization (WHO) [19]. The 39-item questionnaire assesses the occurrence and frequency of 13 distinct types of ACEs: emotional neglect, physical neglect, emotional abuse, physical abuse, sexual abuse, domestic violence, parental loss or separation, living with a household member who has substance abuse, has been incarcerated, or is mentally ill or suicidal, community violence, collective violence, and bullying (Supplementary Table S1). We followed WHO’s frequency scoring system [19] to generate three exposure variables: (i) an ACE total score (ranging from 0 to 13) reflecting the number of ACE types experienced; (ii) a categorical variable where the ACE total score was categorized based on the distribution of the sample into: 0 ACEs, 1 ACE, 2 ACEs, 3–4 ACEs, and 5 or more ACEs; (iii) a binary variable for each ACE type.

### Type 2 diabetes

Two questions were used to calculate a binary indicator for type 2 diabetes. Participants first indicated if they had been diagnosed with diabetes (negative respondents coded as not having type 2 diabetes) and subsequently what type of diabetes they had. Those who reported having type 1 diabetes or who did not report the type of diabetes were excluded from the analysis. Participants were asked how their diabetes was treated with response options including changed diet, oral medication, or insulin injections. Those who responded affirmatively to the latter two options (*n* = 566) were asked whether diabetes had caused further health problems (problems with kidneys, sight, or neither). Participants were asked to indicate their age at the time of the diagnosis and whether they had ever been diagnosed with gestational diabetes. Questions and response options relating to diabetes are presented in Supplementary Fig. S2.

### Covariates

The covariates considered included age and childhood deprivation (potential confounders), as well as adult BMI, smoking, education, income, civil status (potential mediators), current alcohol consumption, and current depressive symptoms (Supplementary Fig. S3). Childhood deprivation was defined as whether their family’s economic hardship led to deprivation (e.g. of nutritious food or warm clothing) and was used as a binary variable of never vs. ever experiencing childhood deprivation. Education was categorized into primary, secondary, BSc or equivalent, and MSc or above. Income was categorized into low, low-medium, medium, high-medium, and high income. Smoking status was categorized into never smoker or ever smoker for statistical analyses. Alcohol consumption was measured by frequency of binge drinking (six or more alcoholic drinks in one sitting) during the last 12 months and was categorized into five categories [20]. Depressive symptoms in the past two weeks were defined as a score of 10 or higher on the Patient Health Questionnaire-9 (PHQ-9) [21]. Covariates are described in greater detail in Supplementary Table S2.

### Data analysis

Descriptive characteristics are presented with numbers and percentages, or means and standard deviations (SD). We used log-linear modified Poisson regression models with robust error variance to quantify the associations between ACEs and type 2 diabetes [22]. ACEs were used both as a continuous and categorical variable with zero ACEs as a reference group and as a binary variable for each ACE subtype. We retained a linear model after testing for the linearity of the relationship between ACEs and type 2 diabetes using natural splines which did not offer significant improvements to the fit (*P* = 0.465). All main analyses were adjusted for age and childhood deprivation. ACE subtype analyses were conducted for each ACE separately and with all ACEs included in the model.

Using the CMAverse package from R version 0.1.0 [23], we carried out a causal mediation analysis to estimate the total effect and total natural direct effect (TNDE) of ACEs on type 2 diabetes, the total natural indirect effect (TNIE), and percentage of the effect mediated collectively through adult BMI, education, income, civil status, and smoking, allowing for exposure mediator interactions. Assuming no unmeasured confounding in the exposure-outcome, mediator-outcome, and exposure-mediator associations nor confounding in the mediator–outcome association by the exposure [23, 24], the analysis was adjusted for age and childhood deprivation. We examined effect modification using models with ACE-covariate interaction terms, adjusting for age and childhood deprivation, and reported subgroup-specific prevalence ratios (PR), using analysis of variance (ANOVA) to test for significance. To further explore the role of lifestyle factors and mental health we reported stratified analyses by binge drinking frequency and depressive symptoms in supplementary models.

The ACE subtype parental loss or separation includes parental loss due to death and parental separation/divorce, a relatively common experience of Icelandic children. Given their conceptual differences, a sensitivity analysis was carried out where we excluded parental separation/divorce from the total ACE score. We carried out analyses to address risk of misclassification of type 2 diabetes due to reporting error, firstly excluding women who had been diagnosed with gestational diabetes and secondly, those who managed their diabetes by changed diet were classified as not having type 2 diabetes. To explore potential confounding of the association between ACE subtypes and type 2 diabetes, we additionally adjusted for adult BMI. The association between ACEs and age at diagnosis of type 2 diabetes, was tested using an ANOVA with age at diagnosis of type 2 diabetes as a continuous outcome, adjusted for childhood deprivation. PRs and 95% confidence intervals (CI) were reported from all modified Poisson regressions.

We used predictive mean matching with *m* = 20 to replace missing values of covariates [25]. Descriptive statistics for women with missing values on any of the covariates are presented in Supplementary Table S3. The auxiliary variables used for imputation are described in Supplementary Table S4. We used row imputation to replace missing values in the PHQ-9 questionnaire for participants with responses to more than 75% of the items. All analyses were conducted using the imputed dataset (*n* = 26 952), except for the mediation analysis, complete case analysis, and models with interaction terms which were conducted after excluding women with missing values for any of the covariates (*n* = 24 823) (Supplementary Fig. S1). R (version 4.2.2) was used for all statistical analyses.

## Results

### Descriptive characteristics

**Figure 1.**
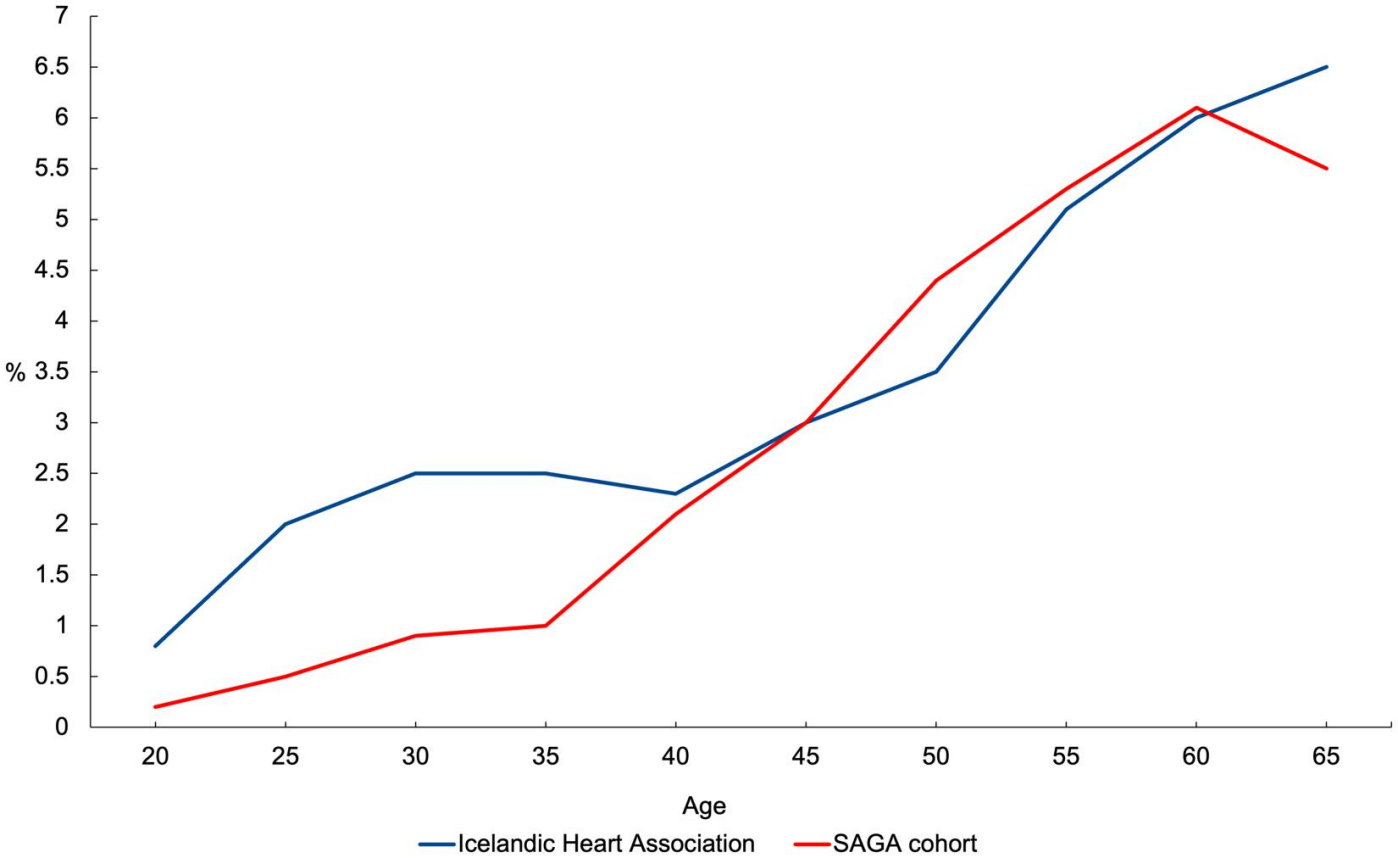
Comparison of self-reported prevalence of type 2 diabetes among women in the SAGA cohort (red) to the Icelandic Heart Association’s published prevalence rates (blue) acquired from prescription registers (Þórsson et al. 2021).

A total of 776 (2.9%) women reported a diagnosis of type 2 diabetes, similar to the 3.2% prevalence reported in a nationwide study using register data [18] (Fig. 1). The prevalence of type 2 diabetes increased with age and was largely similar among women over 40 years in the SAGA cohort and the register-based estimates [18], while somewhat lower among women under 40 years in the SAGA cohort (Fig. 1). The majority of the women with type 2 diabetes were treated with oral medications (62.2%) (Supplementary Table S4). In the sample, 21 429 (79.5%) of the women reported one or more ACEs and 5256 (19.5%) reported five or more ACEs (Table 1). Compared to women with a low burden of ACEs, those with a higher burden of ACEs had, on average, higher BMI, reported childhood deprivation more often, were more likely to be less educated and single, separated, or widowed, to have a lower income, to have smoked and engaged in frequent binge drinking, and to report depressive symptoms (Table 1). Supplementary Table S6 shows descriptive statistics by diagnosis of type 2 diabetes.

**Table 1.** Characteristics of the sample by the number of ACEs reported

	Number of ACEs, *N* (%)					
	0 ACEs	1 ACE	2 ACEs	3–4 ACEs	5+ ACEs	Total
Total	5523	5810	4598	5765	5256	26 952
Age, mean (SD)	43.8 (14.0)	44.9 (14.0)	44.8 (13.7)	44.3 (13.5)	43.2 (12.8)	44.2 (13.6)
BMI, mean (SD)	27.1 (5.5)	27.7 (5.8)	28.0 (5.9)	28.1 (6.0)	28.6 (6.8)	27.9 (6.0)
Age groups						
18-29 years	1130 (20.5)	1042 (17.9)	816 (17.7)	1054 (18.3)	968 (18.4)	5010 (18.6)
30-39 years	1131 (20.5)	1147 (19.7)	875 (19.0)	1120 (19.4)	1118 (21.3)	5391 (20.0)
40-49 years	1157 (20.9)	1207 (20.8)	1018 (22.1)	1312 (22.8)	1336 (25.4)	6030 (22.4)
50-59 years	1170 (21.2)	1339 (23.0)	1120 (24.4)	1397 (24.2)	1224 (23.3)	6250 (23.2)
60-69 years	935 (16.9)	1075 (18.5)	769 (16.7)	882 (15.3)	610 (11.6)	4271 (15.8)
Childhood deprivation						
Never	5196 (94.1)	5130 (88.3)	3704 (80.6)	4016 (69.7)	2311 (44.0)	20 357 (75.5)
Rarely	230 (4.2)	448 (7.7)	524 (11.4)	868 (15.1)	910 (17.3)	1175 (4.4)
Sometimes	77 (1.4)	199 (3.4)	298 (6.5)	647 (11.2)	1150 (21.9)	1175 (4.4)
Often	16 (0.3)	24 (0.4)	62 (1.3)	209 (3.6)	864 (16.4)	2371 (8.8)
Missing	4 (0.1)	9 (0.2)	10 (0.2)	25 (0.4)	21 (0.4)	69 (0.3)
BMI						
<25	2259 (40.9)	2191 (37.7)	1599 (34.8)	1981 (34.4)	1772 (33.7)	9802 (36.4)
25–30	1801 (32.6)	1870 (32.2)	1512 (32.9)	1862 (32.3)	1588 (30.2)	8633 (32.0)
30+	1391 (25.2)	1674 (28.8)	1441 (31.3)	1837 (31.9)	1834 (34.9)	8177 (30.3)
Missing	72 (1.3)	75 (1.3)	46 (1.0)	85 (1.5)	62 (1.2)	340 (1.3)
Education						
Primary	501 (9.1)	731 (12.6)	616 (13.4)	918 (15.9)	1116 (21.2)	3882 (14.4)
Secondary	1519 (27.5)	1725 (29.7)	1397 (30.4)	1809 (31.4)	1820 (34.6)	8270 (30.7)
BSc or equivalent	1976 (35.8)	1938 (33.4)	1521 (33.1)	1791 (31.1)	1351 (25.7)	8577 (31.8)
MSc or above	1513 (27.4)	1398 (24.1)	1042 (22.7)	1230 (21.3)	934 (17.8)	6117 (22.7)
Missing	14 (0.3)	18 (0.3)	22 (0.5)	17 (0.3)	35 (0.7)	106 (0.4)
Monthly income						
Low income	1309 (23.7)	1609 (27.7)	1254 (27.3)	1801 (31.2)	1979 (37.7)	7952 (29.5)
Low-medium income	1593 (28.8)	1745 (30.0)	1428 (31.1)	1750 (30.4)	1583 (30.1)	8099 (30.0)
Medium income	1448 (26.2)	1408 (24.2)	1106 (24.1)	1253 (21.7)	992 (18.9)	6207 (23.0)
High-medium income	675 (12.2)	585 (10.1)	477 (10.4)	541 (9.4)	405 (7.7)	2683 (10.0)
High income	261 (4.7)	229 (3.9)	146 (3.2)	201 (3.5)	115 (2.2)	952 (3.5)
Missing	237 (4.3)	234 (4.0)	187 (4.1)	219 (3.8)	182 (3.5)	1059 (3.9)
Civil status						
Married/in relationship	4359 (78.9)	4505 (77.5)	3508 (76.3)	4261 (73.9)	3699 (70.4)	20 332 (75.4)
Single or widowed	1142 (20.7)	1288 (22.2)	1072 (23.3)	1468 (25.5)	1511 (28.7)	6481 (24.0)
Missing	22 (0.4)	17 (0.3)	18 (0.4)	36 (0.6)	46 (0.9)	139 (0.5)
Smoking						
Never	3471 (62.8)	3159 (54.4)	2173 (47.3)	2320 (40.2)	1591 (30.3)	12 714 (47.2)
Previous smoker	1507 (27.3)	1876 (32.3)	1699 (37.0)	2348 (40.7)	2361 (44.9)	9791 (36.3)
Yes, but not daily	245 (4.4)	321 (5.5)	275 (6.0)	395 (6.9)	342 (6.5)	1578 (5.9)
Yes, daily	233 (4.2)	380 (6.5)	402 (8.7)	637 (11.0)	900 (17.1)	2552 (9.5)
Missing	67 (1.2)	74 (1.3)	49 (1.1)	65 (1.1)	62 (1.2)	317 (1.2)
Binge drinking						
Never	2657 (48.1)	2733 (47.0)	2092 (45.5)	2579 (44.7)	2555 (48.6)	12 616 (46.8)
Less than once a month	2179 (39.5)	2265 (39.0)	1822 (39.6)	2214 (38.4)	1824 (34.7)	10 304 (38.2)
Monthly	502 (9.1)	568 (9.8)	475 (10.3)	647 (11.2)	518 (9.9)	2710 (10.1)
Weekly	134 (2.4)	157 (2.7)	143 (3.1)	212 (3.7)	237 (4.5)	883 (3.3)
Daily or almost daily	4 (0.1)	22 (0.4)	14 (0.3)	21 (0.4)	50 (1.0)	111 (0.4)
Missing	47 (0.9)	65 (1.1)	52 (1.1)	92 (1.6)	72 (1.4)	328 (1.2)
Depressive symptoms						
No	4736 (85.8)	4536 (78.1)	3294 (71.6)	3642 (63.2)	2444 (46.5)	18 652 (69.2)
Yes	768 (13.9)	1241 (21.4)	1282 (27.9)	2094 (36.3)	2791 (53.1)	8176 (30.3)
Missing	19 (0.3)	33 (0.6)	22 (0.5)	29 (0.5)	21 (0.4)	124 (0.5)

### Associations between ACEs and type 2 diabetes

**Figure 2.**
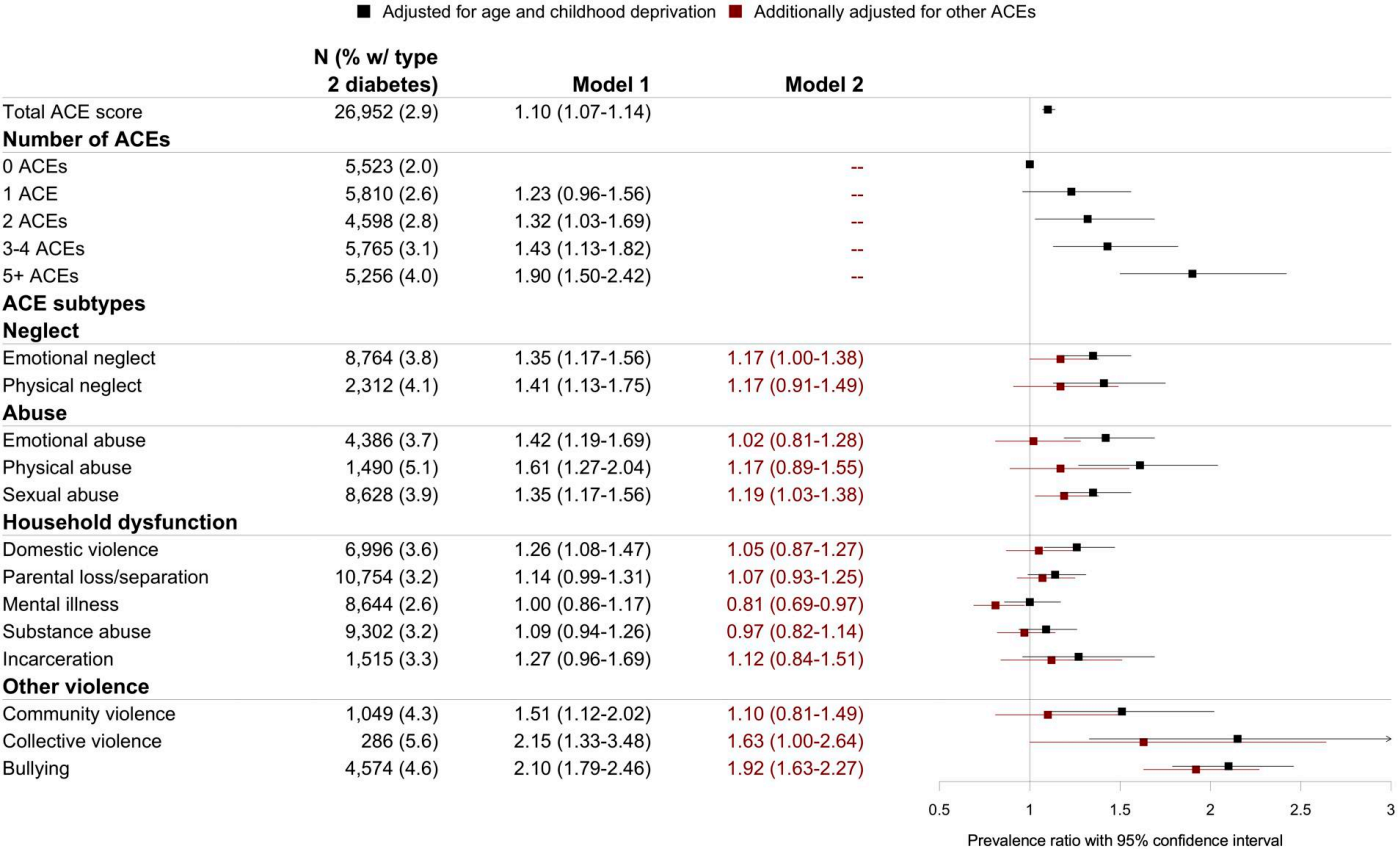
Prevalence of type 2 diabetes by number and subtype of ACEs and the association between ACEs and type 2 diabetes (prevalence ratio and 95% CI). Adjusted for age and childhood deprivation (model 1) and additionally for other ACEs (model 2).

The prevalence of type 2 diabetes increased with the number of ACEs (Fig. 2), and log-linear Poisson regression models showed a stronger association between ACEs and type 2 diabetes as the number of ACEs increased. Women who reported five or more ACEs had double the prevalence of type 2 diabetes (PR 1.90 [1.50–2.42]) compared to those with no ACEs. Each additional ACE reported corresponded to an 10% (1.07–1.14) increase in the prevalence of type 2 diabetes. Sensitivity analyses showed slightly stronger associations after excluding parental separation from the ACE total score (Supplementary Table S7), and similar results after excluding gestational diabetes (Supplementary Table S8) or diabetes managed by a changed diet (Supplementary Table S9). The complete case analysis yielded similar results as the main model (Supplementary Table S10). The gradient increase in the association by number of ACEs was stable throughout all models. The association between ACEs and type 2 diabetes was found to be significant among those who engaged in binge drinking less than monthly or never and remained significant irrespective of current depressive symptoms (Supplementary Table S11).

All ACE categories, except parental loss/separation and growing up with a household member with substance abuse, incarceration, or mental illness, were associated with type 2 diabetes (Fig. 2). When mutually adjusting the ACEs for one another the following remained statistically significant: bullying (PR 1.92 [1.63–2.27]), sexual abuse (PR 1.19 [1.03–1.38]), and mental illness of a household member (PR 0.81 [0.69–0.97]). When additionally adjusting for adult BMI, the association between bullying and type 2 diabetes diminished somewhat but remained significant (PR 1.62 [1.37–1.90]) (Supplementary Fig. S4).

Causal mediation analysis revealed that women with five or more ACEs had 64% increased odds of type 2 diabetes compared to those without a history of ACEs (TNDE OR [odds ratio] 1.64 [1.25–2.26]), independent of the collective effects of adult BMI, smoking, education, income, and civil status. The total effect of ACEs was 2.30 (1.76–3.15) but a significant association between ACEs and type 2 diabetes was mediated through these mediators (TNIE OR 1.25 [1.11–1.41]) which collectively explained 35.3% (16.5–53.6%) of the association. We tested exposure–mediator associations and found ACEs to be significantly associated with all mediators (Supplementary Table S12). Subgroup-specific associations between ACEs and type 2 diabetes remained significant across most subgroups, and no significant ACE-covariate interaction was found (Supplementary Fig. S5).

The mean age of diagnosis of type 2 diabetes in the sample was 46.9 years. ACEs were significantly associated with age at diagnosis (*F*[4, 769] =5.92; *P* < 0.001). Women with five or more ACEs were diagnosed younger than those with 0 ACEs (−4.4 years; *P* = 0.006), 1 ACE (−4.3 years; *P* = 0.002), 2 ACEs (−5.7 years; *P* < 0.001) and 3–4 ACEs (−3.2 years; *P* = 0.03) (Supplementary Fig. S6). Women with a higher burden of ACEs were more likely to use insulin injections to treat their type 2 diabetes (Supplementary Table S5).

## Discussion

In this nationwide-representative study of women in Iceland, we found that an accumulation of ACEs was associated with a higher prevalence of type 2 diabetes among adult women. Women who reported five or more ACEs had double the prevalence of type 2 diabetes compared to women with no ACEs, after accounting for confounders. ACEs were directly associated with type 2 diabetes independently of adult BMI, smoking, and socioeconomic factors, yet these factors were found to explain 35% of the association between ACEs and type 2 diabetes. Of the 13 types of ACEs, we found that bullying and sexual abuse presented the most robust independent associations with type 2 diabetes.

The findings of the present study are in accordance with the greater body of existing literature [3, 4, 6, 7, 10, 26–28]. Nevertheless, some studies have reported no significant association between ACEs and diabetes [8, 11, 12] while others have reported stronger associations among men than women, or have found significant associations exclusively among men [9, 13, 14, 29]. A recent meta-analysis of 17 studies reported an overall association between ACEs and diabetes, with significant associations found among men but not women [9]. However, many of these studies, including the meta-analysis, focus on individual ACEs rather than a cumulation of multiple types of ACEs in their reporting of sex-aggregated results. Previous research [1, 7] underscores the importance of focusing on cumulative ACEs to explore the comprehensive impact of ACEs on adult health.

Subgroup-specific associations between ACEs and type 2 diabetes were robust across almost all subgroups of women. Existing literature has indicated stronger associations between ACEs and type 2 diabetes among young adults, compared to older adults [26, 27]. Our findings did not confirm those findings, but we did however find that women with a higher burden of ACEs were diagnosed with type 2 diabetes at a significantly earlier age compared to women without ACEs.

The nature and severity of ACE subtypes may contribute to the varying strength of association with type 2 diabetes. Some studies have reported strong associations between neglect and type 2 diabetes while others have observed strong associations with sexual abuse [6, 9, 10, 12], of which the latter was independently associated with type 2 diabetes in the present study. The comprehensive range of ACEs measured in our study allowed us to assess the role of bullying, for which the association to type 2 diabetes has not been studied previously, to the best of our knowledge. Bullying was found to be the ACE subtype with the strongest association with type 2 diabetes, with a two-fold higher prevalence of type 2 diabetes among women exposed to bullying. Although the association attenuated somewhat when adjusted for adult BMI, bullying remained strongly associated with type 2 diabetes.

The similarities in the prevalence of self-reported type 2 diabetes in the SAGA cohort and a previous study using national prescription data [18] lend further support to the representativeness of the SAGA cohort population. In women under 40 years however, the prevalence was relatively lower in the SAGA cohort. This could, in part, be attributed to medications, such as metformin [30], being used for other purposes than to treat type 2 diabetes among women of a reproductive age. The comparison of the studies is somewhat limited, as some women classified as having type 2 diabetes in the SAGA cohort report managing their condition through dietary changes. The questionnaire lacks specificity regarding whether they exclusively managed their diabetes this way (Supplementary Fig. S2) but if they had, they would not appear in the prescription registers.

### Possible mechanisms for the link between adverse childhood experiences and type 2 diabetes

Chronic stress in childhood, resulting depression, and the development of unhealthy behaviors have been suggested as possible pathways linking ACEs and type 2 diabetes. Chronic stress leaves children vulnerable to impaired development of the nervous, immune, and endocrine systems [31, 32] and can elevate the activity of the hypothalamic–pituitary adrenal axis, promoting secretion of hormones that impair insulin receptor signaling [33]. Together these factors may contribute to elevated blood glucose levels [34] and increase the risk of type 2 diabetes [35, 36]. Furthermore, ACEs are predictors for known risk factors of type 2 diabetes such as a sedentary lifestyle, poor diet, and alcohol abuse [6, 8, 37].

Our results from the causal mediation analysis align to some extent with previous research. Wen et al. [16] found BMI, high density lipoproteins, mental health, smoking and socioeconomic status to be among the primary mediators of the association between childhood maltreatment and type 2 diabetes. Similarly, Deschênes et al. [15] reported depression and cardiometabolic dysregulation to mediate the association between ACEs and diabetes, finding no direct association independent of these factors. Other research has suggested sleep duration and exposure to violence in adulthood as potential mediating pathways [38, 39]. In the current study, we found adult BMI, smoking, education, income, and civil status to collectively mediate 35% of the association although ACEs were also directly associated with type 2 diabetes. Binge drinking (in the preceding 12 months) and depressive symptoms (in the preceding two weeks) were not included in the mediation analysis as they were measured based on their status at the time of data collection and were regarded to be susceptible to change after a diagnosis of type 2 diabetes.

### Strengths and limitations

Strengths of the present study include the large sample size of the SAGA cohort, its nationwide-representative design, and the representativeness of the sample [17]. The study included a broad spectrum of childhood adversities, allowing to measure commonly overlooked ACEs such as bullying, as well as an accumulation of ACEs. Additionally, we differentiated between type 2 and type 1 diabetes, an advantage lacking in many previous studies. Finally, despite its cross-sectional design, there is built-in temporality of ACEs and type 2 diabetes, mitigating concerns of reverse causality.

The limitations of the study entail its cross-sectional design which restrict inferences on the association, particularly on the effects of mediation. Covariates were reported based on their status at the time and may not accurately indicate their presence in the causal pathway. Moreover, the assumptions of the mediation analysis are not fully verifiable and, if violated, may introduce bias to the estimates. Analysing the mediators collectively, however, is more robust toward unmeasured confounding [24]. The strong association bullying has with type 2 diabetes may be partly explained by unmeasured confounding such as childhood BMI. The association remained significant when adjusting for adult BMI, although to what extent adult BMI reflects childhood BMI or serves as a mediator in this context remains unknown. While the sample is representative of the Icelandic female population in terms of income, residence, education, and age, there remains some minor underrepresentation of younger and less educated populations. As our study included women exclusively, inferences cannot be made about men and gender minorities. Similarly, the study was conducted in Iceland, a high-income country with a small population. Factors influencing the prevalence of ACEs, type 2 diabetes, and their association may differ across settings, limiting the generalizability of our findings, particularly outside of high-income countries. Finally, as ACEs were reported retrospectively and all variables were reported simultaneously—differential misclassification of ACEs may arise if women with type 2 diabetes have had more opportunity to reflect on their health status or have more negative response tendencies—which may lead to inflated effect estimates. Nevertheless, ACEs are rarely reported to authorities and self-reporting is considered the most accurate way of assessing ACEs [40]. Additionally, adjusting for depressive symptoms may capture mental health status at the time of reporting which could have influenced participants’ answers.

To conclude, our results reiterate the heavy burden and health consequences related to an accumulation of adversity during childhood. We observed a dose–response association between the number of ACEs endorsed and the prevalence of type 2 diabetes among Icelandic women in a nationwide, representative cohort. This association was to some extent but not entirely explained by adult factors such as BMI, smoking, and socioeconomic status. Sexual abuse during childhood was found to be independently associated with type 2 diabetes in adulthood and so was bullying, an association that, to our knowledge, has not been explored in previous studies. While future research, ideally with prospective design, is warranted to advance our understanding of the underlying mechanisms of the association between ACEs and type 2 diabetes, these findings further highlight the importance of careful monitoring of the adult health consequences of ACEs.

## Supplementary Material

ckaf079_Supplementary_Data

## Data Availability

As the datasets used in this study are subject to ethical approval of the National Bioethics Committee (NBC) of Iceland they cannot be shared publicly under Icelandic data protection law. To access the data, interested researchers can submit a proposal to the SAGA cohort data management board which can assist in obtaining approval from the NBC.

## Prior presentation statement

Preliminary results from this study were presented at the annual meeting of the Scandinavian Society for the Study of Diabetes in Reykjavík on 13 May 2022, the 10th Nordic conference on epidemiology and register-based research (NordicEpi) in Reykjavík on 19 August 2022, and at an educational event for nurses working with diabetes in Reykjavík on 6 December 2022. Preliminary results of this manuscript were included in a bachelor’s thesis available in the Icelandic digital repository of academic and research documents, Skemman.

Key points

- We found a dose–response relationship between the number of adverse childhood experiences and prevalence of type 2 diabetes among adult women, where women with five or more ACEs had double the prevalence of type 2 diabetes compared to those reporting no ACEs, after adjusting for confounders.
- The association is to some extent mediated collectively by adult BMI, smoking, and socioeconomic factors, but remains significant independent of these factors.
- Bullying and sexual abuse in childhood were independently found to be positively associated with type 2 diabetes among adult women, of which bullying presented the strongest association.
- Addressing the impact of adverse childhood experiences in the prevention and care of type 2 diabetes can help identify high-risk individuals and inform interventions at both the patient level and through public health strategies.
