# Supplementary material for: Adverse childhood experiences and prevalence of type 2 diabetes in a nationwide study of women
Source: Eur J Public Health. 2025 Jun 10;35(5):882–8. doi: 10.1093/eurpub/ckaf079 (PMC12529292; doi:10.1093/eurpub/ckaf079)

**Adverse childhood experiences and prevalence of type 2 diabetes in a nationwide study of women**

Elísabet U. Gísladóttir, Hilda B. Daníelsdóttir, Huan Song, Marín D. Bjarnardóttir, Arna Hauksdóttir, Arna Guðmundsdóttir, Diego Yacamán Méndez, Edda B. Bjork Thordardottir, Gunnar Tomasson, Harpa Rúnarsdóttir, Jóhanna Jakobsdóttir, Fang Fang, Unnur A. Valdimarsdóttir, and Thor Aspelund

**Table of Contents**

[Figure S1. Flowchart of sample selection 2](#_Toc197900125)

[Table S1. Items and response options on the ACE-IQ 2](#_Toc197900126)

[Figure S2. Flowchart of questions and response options relating to diabetes 4](#_Toc197900127)

[Figure S3. Hypothesized causal model of how ACEs could influence the development of type 2 diabetes in adulthood^*^ 4](#_Toc197900128)

[Table S2. Covariate details* 4](#_Toc197900129)

[Table S3. Characteristics of the imputed sample and participants with missing values on any covariates 5](#_Toc197900130)

[Table S4. List of auxiliary variables for multiple imputation 6](#_Toc197900131)

[Table S5. Clinical characteristics of women with type 2 diabetes 6](#_Toc197900132)

[Table S6. Descriptive characteristics by number of ACEs 7](#_Toc197900133)

[Table S7. Association between ACEs and type 2 diabetes excluding parental divorce or separation as an ACE (prevalence ratio and 95% CI) 8](#_Toc197900134)

[Table S8. Association between ACEs and type 2 diabetes excluding women ever diagnosed with gestational diabetes (prevalence ratio and 95% CI)^*^ 8](#_Toc197900135)

[Table S9. Association between ACEs and type 2 diabetes where women who managed diabetes by a changed diet were classified as not having type 2 diabetes (prevalence ratio and 95% CI) 9](#_Toc197900136)

[Table S10. Association between ACEs and type 2 diabetes in a complete dataset (prevalence ratio and 95% CI)^*^ 9](#_Toc197900137)

[Table S11. Association between ACEs and type 2 diabetes stratified by binge drinking frequency and depressive symptoms (prevalence ratio and 95% CI) 9](#_Toc197900138)

[Figure S4. Individual ACE categories adjusted for age, childhood deprivation and BMI 10](#_Toc197900139)

[Figure S5. Subgroup-specific associations between total ACE score and type 2 diabetes, including ACE–covariate interaction terms for each covariate (PR and 95% CI)^*^ 11](#_Toc197900140)

[Figure S6. Means and standard deviation of age at diagnosis of type 2 diabetes by number of ACEs. Each dot represents one woman with type 2 diabetes in the analytic sample. 12](#_Toc197900141)

# Figure S1. Flowchart of sample selection


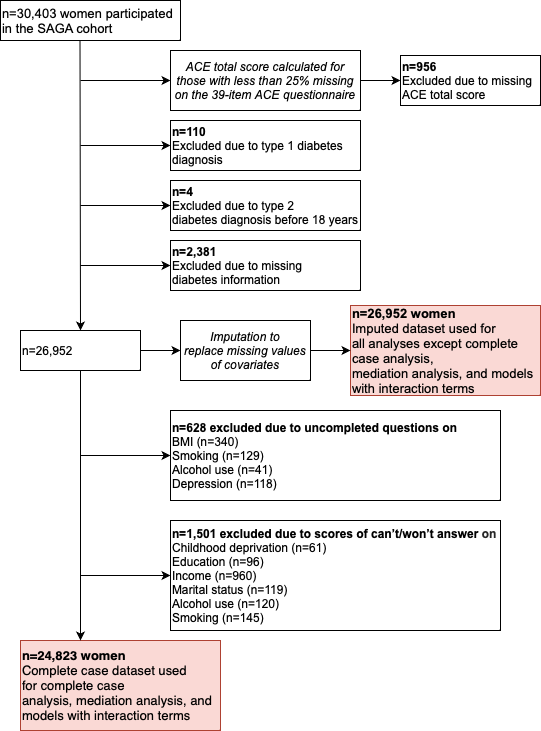


# Table S1. Items and response options on the ACE-IQ

| **Neglect** |  |
| --- | --- |
| **Emotional neglect** |  |
| Did your parents/guardians understand your problems and worries? | Always = 0, Most of the time = 1, Sometimes = 2, Rarely = 3, Never = 4, Can’t/won’t answer = 5 |
| Did your parents/guardians really know what you were doing with your free time when you were not at school or work? | Always = 0, Most of the time = 1, Sometimes = 2, Rarely = 3, Never = 4, Can’t/won’t answer = 5 |
| **Physical neglect** |  |
| How often did your parents/guardians not give you enough food even when they could easily have done so? | Never = 0, Once = 1, A few times = 2, Many times = 3, Can’t/won’t answer = 4 |
| Were your parents/guardians too drunk or intoxicated by drugs to take care of you? | Never = 0, Once = 1, A few times = 2, Many times = 3, Can’t/won’t answer = 4 |
| How often did your parents/guardians not send you to school even when it was available? | Never = 0, Once = 1, A few times = 2, Many times = 3, Can’t/won’t answer = 4 |
| **Abuse** |  |
| **Emotional abuse** |  |
| Did a parent, guardian or other household member yell, scream or swear at you, insult or humiliate you? | Never = 0, Once = 1, A few times = 2, Many times = 3, Can’t/won’t answer = 4 |
| Did a parent, guardian or other household member threaten to, or really abandon you or throw you out of the house? | Never = 0, Once = 1, A few times = 2, Many times = 3, Can’t/won’t answer = 4 |
| **Physical abuse** |  |
| Did a parent, guardian or other household member spank, slap, kick, punch or beat you up? | Never = 0, Once = 1, A few times = 2, Many times = 3, Can’t/won’t answer = 4 |
| Did a parent, guardian or other household member hit or cut you with an object, such as a stick (or cane), bottle, club, knife, whip etc.? | Never = 0, Once = 1, A few times = 2, Many times = 3, Can’t/won’t answer = 4 |
| **Sexual abuse** |  |
| Did someone touch or fondle you in a sexual way when you did not want them to? | Never = 0, Once = 1, A few times = 2, Many times = 3, Can’t/won’t answer = 4 |
| Did someone make you touch their body in a sexual way when you did not want them to? | Never = 0, Once = 1, A few times = 2, Many times = 3, Can’t/won’t answer = 4 |
| Did someone attempt oral, anal, or vaginal intercourse with you when you did not want them to? | Never = 0, Once = 1, A few times = 2, Many times = 3, Can’t/won’t answer = 4 |
| Did someone have oral, anal, or vaginal intercourse with you when you did not want them to? | Never = 0, Once = 1, A few times = 2, Many times = 3, Can’t/won’t answer = 4 |
| **Household dysfunction** |  |
| **Domestic violence** |  |
| Did you see or hear a parent or household member in your home being yelled at, screamed at, sworn at, insulted or humiliated? | Never = 0, Once = 1, A few times = 2, Many times = 3, Can’t/won’t answer = 4 |
| Did you see or hear a parent or household member in your home being slapped, kicked, punched or beaten up? | Never = 0, Once = 1, A few times = 2, Many times = 3, Can’t/won’t answer = 4 |
| Did you see or hear a parent or household member in your home being hit or cut with an object, such as a stick (or cane), bottle, club, knife, whip etc.? | Never = 0, Once = 1, A few times = 2, Many times = 3, Can’t/won’t answer = 4 |
| **Lost a parent / parental separation** |  |
| Were your parents ever separated or divorced? | No = 0, Yes = 1, Can’t/won’t answer = 2 |
| Did your mother, father or guardian die? | No = 0, Yes = 1, Can’t/won’t answer = 2 |
| **Mental illness in household** |  |
| Did you live with a household member who was depressed, mentally ill or suicidal? | No = 0, Yes = 1, Can’t/won’t answer = 2 |
| **Household substance abuse** |  |
| Did you live with a household member who was a problem drinker or alcoholic, or misused street or prescription drugs? | No = 0, Yes = 1, Can’t/won’t answer = 2 |
| **Incarcerated household member** |  |
| Did you live with a household member who was ever sent to jail or prison? | No = 0, Yes = 1, Can’t/won’t answer = 2 |
| **Other violence** |  |
| **Community violence** |  |
| Did you see or hear someone being beaten up in real life? | Never = 0, Once = 1, A few times = 2, Many times = 3, Can’t/won’t answer = 4 |
| Did you see or hear someone being stabbed or shot in real life? | Never = 0, Once = 1, A few times = 2, Many times = 3, Can’t/won’t answer = 4 |
| Did you see or hear someone being threatened with a knife or gun in real life? | Never = 0, Once = 1, A few times = 2, Many times = 3, Can’t/won’t answer = 4 |
| **Collective violence** |  |
| During the first 18 years of your life, were you exposed to war/collective violence (e.g. from gangs or police)?* | Never = 0, Once = 1, A few times = 2, Many times = 3, Can’t/won’t answer = 4 |
| Were you forced to go and live in another place due to any of these events? | Never = 0, Once = 1, A few times = 2, Many times = 3, Can’t/won’t answer = 4 |
| Did you experience the deliberate destruction of your home due to any of these events? | Never = 0, Once = 1, A few times = 2, Many times = 3, Can’t/won’t answer = 4 |
| Were you beaten up by soldiers, police, militia, or gangs? | Never = 0, Once = 1, A few times = 2, Many times = 3, Can’t/won’t answer = 4 |
| Was a family member or friend killed or beaten up by soldiers, police, militia, or gangs? | Never = 0, Once = 1, A few times = 2, Many times = 3, Can’t/won’t answer = 4 |
| **Bullying** |  |
| How often were you bullied? | Never = 0, Once = 1, A few times = 2, Many times = 3, Can’t/won’t answer = 4 |

*This question was added as a screening question – those who responded negatively were not asked the following questions on collective violence.

#
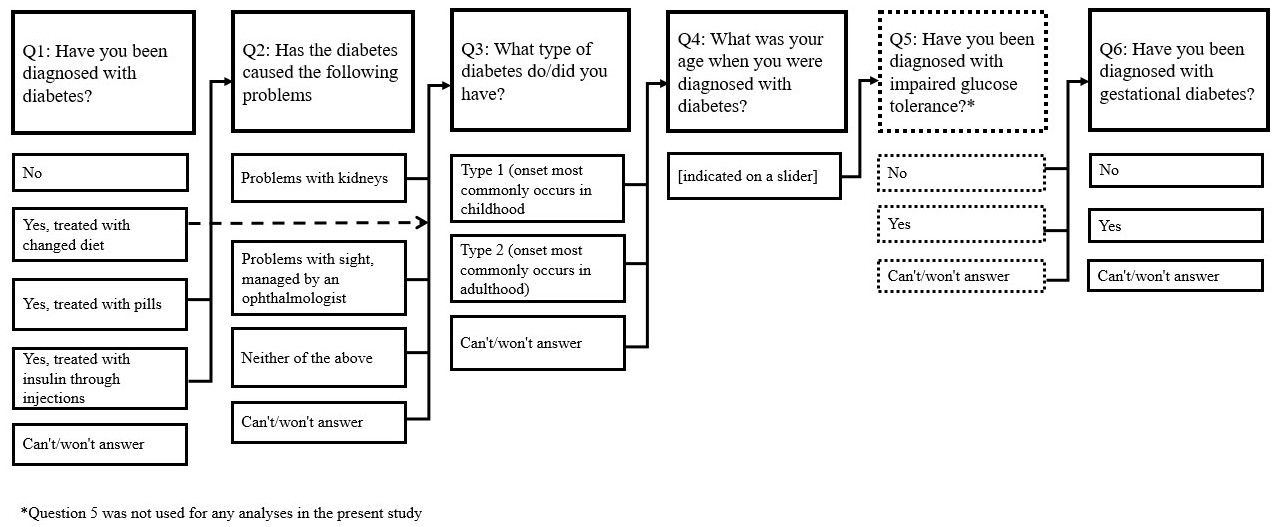
Figure S2. Flowchart of questions and response options relating to diabetes

# Figure S3. Hypothesized causal model of how ACEs could influence the development of type 2 diabetes in adulthood^*^

^*^Orange box indicates potential confounders, green box indicates potential mediators and purple box indicates potential colliders.

# Table S2. Covariate details*

| **Age** |
| --- |
| Age was categorized into five groups (18-29 years, 30-39 years, 40-49 years, 50-59 years, and over 60 years) for model adjustments and descriptive analysis. When including interaction terms, the two youngest age groups were joined (18-39 years). |
| **Childhood deprivation** |
| Childhood deprivation reflected how often in their childhood the family‘s economic situation was so poor that they were unable to afford necessities such as food and warm clothing. Childhood deprivation was used as a binary variable (no childhood deprivation or any deprivation) for all analyses except for descriptive purposes where all original categories (Never, rarely, sometimes and often) were demonstrated. |
| **Body mass index** |
| Body mass index (BMI) was calculated from self-reported height and weight and was used as a categorical variable (under 25 kg/m^2^, 25-30 kg/m^2^, and over 30 kg/m^2^). |
| **Education** |
| Education level was defined as highest educational achievement and was categorized into primary education, secondary education, tertiary education A (BSc or equivalent), and tertiary education B (MSc or above). |
| **Income** |
| Income was defined by monthly personal income and was categorized into low income (<$2527 ), low-medium income ($2528- $4212), medium income ($4213-$5897), high-medium income ($5898-$8424), and high income (>$8425; conversion rates from the Central Bank of Iceland at the time of the data collection). When including interaction terms, the two highest income groups were joined (>$5898). |
| **Civil status** |
| Civil status included two categories of married or in a relationship and single, divorced or widowed. |
| **Smoking status** |
| Smoking status was used as a binary variable or never smoked and ever smoked for all analyses except for descriptive purposes (never smoked, previous smoker, current non-daily smoker and current daily smoker) |
| **Alcohol consumption** |
| Alcohol consumption was measured using the third item of the Alcohol Use Disorders Identification Test, frequency of binge drinking (6 or more alcoholic drinks in one sitting). Participants were asked about their habits in the preceding 12 months. Binge drinking was classified into the following categories: never, less than once a month, monthly, weekly, and daily or almost daily. When including interaction terms, the groups of weekly and daily or almost daily were joined (weekly or more). |
| **Depressive symptoms** |
| Past two-week depressive symptoms were assessed with the Patient Health Questionnaire-9. A cut-off score of 10 was used, which is indicative of clinically significant symptomology. |

*The response option of “can’t/won’t answer” was available for all covariates but age.

# Table S3. Characteristics of the imputed sample and participants with missing values on any covariates

|  | **Participants with missing values** | **Total analytical sample** |
| --- | --- | --- |
| Total | 2,129 | 26,952 |
| Age, mean (SD) | 44.7 (14.3) | 44.2 (13.6) |
| BMI, mean (SD) | 27.1 (6.0) | 27.9 (6.0) |
| **Age groups** |  |  |
| 18-29 years | 427 (20.1) | 5,010 (18.6) |
| 30-39 years | 349 (16.4) | 5,391 (20.0) |
| 40-49 years | 425 (20.0) | 6,030 (22.4) |
| 50-59 years | 542 (25.5) | 6,250 (23.2) |
| 60-69 years | 386 (18.1) | 4,271 (15.8) |
| **Childhood deprivation (n=69 missing)** |  |  |
| Never | 1,548 (75.1) | 20,357 (75.7) |
| Rarely | 226 (11.0) | 2,980 (11.1) |
| Sometimes | 185 (9.0) | 2,371 (8.8) |
| Often | 101 (4.9) | 1,175 (4.4) |
| **BMI (n=340 missing)** |  |  |
| < 25 | 744 (41.6) | 9,802 (36.8) |
| 25-30 | 600 (33.5) | 8,633 (32.4) |
| 30+ | 445 (24.9) | 8,177 (30.7) |
| **Education (n=106 missing)** |  |  |
| Primary | 347 (16.3) | 3,882 (14.4) |
| Secondary | 744 (34.9) | 8,270 (30.7) |
| Tertiary A (BSc or equivalent) | 594 (27.9) | 8,577 (31.8) |
| Tertiary B (MSc or above) | 338 (15.9) | 6,117 (22.7) |
| **Monthly income (n=1,059 missing)** |  |  |
| Low income | 429 (40.1) | 7,952 (30.7) |
| Low-medium income | 328 (30.7) | 8,099 (31.3) |
| Medium income | 219 (20.5) | 6,207 (24.0) |
| High-medium income | 74 (6.9) | 2,683 (10.4) |
| High income | 20 (1.9) | 952 (3.7) |
| **Civil status (n=139 missing)** |  |  |
| Married or in a relationship | 1,521 (76.4) | 20,332 (75.8) |
| Single or widowed | 469 (23.6) | 6,481 (24.2) |
| **Smoking (n=317 missing)** |  |  |
| Never | 848 (46.8) | 12,714 (47.7) |
| Previous smoker | 644 (35.5) | 9,791 (36.8) |
| Yes, but not daily | 107 (5.9) | 1,578 (5.9) |
| Yes, daily | 213 (11.8) | 2,552 (9.6) |
| **Binge drinking (n=328 missing)** |  |  |
| Never | 947 (52.6) | 12,616 (47.4) |
| Less than once a month | 612 (34.0) | 10,304 (38.7) |
| Monthly | 181 (10.5) | 2,710 (10.2) |
| Weekly | 50 (2.8) | 883 (3.3) |
| Daily or almost daily | 11 (0.6) | 111 (0.4) |
| **Depressive symptoms (n=124 missing)** |  |  |
| No | 1,307 (65.2) | 18,652 (69.5) |
| Yes | 698 (34.8) | 8,176 (30.5) |

# Table S4. List of auxiliary variables for multiple imputation

| **Auxiliary variables** |
| --- |
| Location of birth (Nordic vs. not Nordic), residence by health district, employment status, working hours, number of children, frequency of worst lifetime trauma, Life Event Checklist for DSM-5 (LEC-5), past two week symptoms of anxiety (GAD-7), somatic symptom severity (PHQ-15), Premenstrual Dysphoric Disorder symptoms from DSM-5, PTSD related sleep problems (PSQI Addendum for PTSD), past month posttraumatic stress symptoms (PCL-5), past month sleep problems (PSQI), coping ability (CD-RISC). |

# Table S5. Clinical characteristics of women with type 2 diabetes

| **Age at diagnosis, mean (SD)** | 46.7 (11.2) |
| --- | --- |
| **Age at diagnosis by number of ACEs, mean (SD)** |  |
| 0 ACEs | 48.1 (11.4) |
| 1 ACE | 48.1 (10.6) |
| 2 ACEs | 49.4 (10.7) |
| 3-4 ACEs | 46.9 (10.5) |
| 5+ ACEs | 43.7 (11.0) |
| **Treatment of type 2 diabetes, n (%)** |  |
| Diet | 210 (26.9) |
| Oral medication | 485 (62.2) |
| Insulin injections | 85 (10.9) |
| **Insulin usage by number of ACEs, n (%)** |  |
| 0 ACEs | 8 (7.3) |
| 1 ACE | 16 (10.5) |
| 2 ACEs | 14 (10.8) |
| 3-4 ACEs | 16 (9.1) |
| 5+ ACEs | 31 (14.9) |
| **Health problems caused by type 2 diabetes (n=570), n (%)** |  |
| Problems with kidneys | 26 (4.5) |
| Problems with eyesight | 56 (9.8) |
| Neither | 472 (82.9) |
| Can’t/won’t answer | 16 (2.8) |
| **Ever had gestational diabetes, n (%)** |  |
| Yes | 206 (26.4) |
| No | 568 (72.8) |
| Can’t/won’t answer | 6 (0.8) |

# Table S6. Descriptive characteristics by number of ACEs

|  | **Type 2 diabetes, N (%)** | |  |
| --- | --- | --- | --- |
|  | **No** | **Yes** | **Total** |
| Total | 26,176 (97.1) | 776 (2.9) | 26,952 |
| Age, mean (SD) | 43.9 (13.6) | 53.8 (9.9) | 44.2 (13.6) |
| BMI, mean (SD) | 27.7 (5.9) | 33.0 (6.4) | 27.9 (6.0) |
| **Age groups** |  |  |  |
| 18-29 years | 4,993 (99.7) | 17 (0.3) | 5,010 |
| 30-39 years | 5,339 (99.0) | 52 (1.0) | 5,391 |
| 40-49 years | 5,877 (97.5) | 153 (2.5) | 6,030 |
| 50-59 years | 5,947 (95.2) | 303 (4.8) | 6,250 |
| 60-69 years | 4,020 (94.1) | 251 (5.9) | 4,271 |
| **Childhood deprivation** |  |  |  |
| Never | 19,828 (97.4) | 529 (2.6) | 20,357 |
| Rarely | 2,886 (96.8) | 94 (3.2) | 2,980 |
| Sometimes | 2,272 (95.8) | 99 (4.2) | 2,371 |
| Often | 1,123 (95.6) | 52 (4.4) | 1,175 |
| Missing | 67 (97.1) | 2 (2.9) | 69 |
| **BMI** |  |  |  |
| < 25 | 9,746 (99.4) | 56 (0.6) | 9,802 |
| 25-30 | 8,447 (97.8) | 186 (2.2) | 8,633 |
| 30+ | 7,661 (93.7) | 516 (6.3) | 8,177 |
| Missing | 322 (94.7) | 18 (5.3) | 340 |
| **Education** |  |  |  |
| Primary | 3,680 (94.8) | 202 (5.2) | 3,882 |
| Secondary | 7,975 (96.4) | 295 (3.6) | 8,270 |
| Tertiary A (BSc or equivalent) | 8,397 (97.9) | 180 (2.1) | 8,577 |
| Tertiary B (MSc or above) | 6,024 (98.5) | 93 (1.5) | 6,117 |
| Missing | 100 (94.3) | 6 (5.7) | 106 |
| **Monthly income** |  |  |  |
| Low income | 7,663 (96.4) | 289 (3.6) | 7,952 |
| Low-medium income | 7,841 (96.8) | 258 (3.2) | 8,099 |
| Medium income | 6,066 (97.7) | 141 (2.3) | 6,207 |
| High-medium income | 2,639 (98.4) | 44 (1.6) | 2,683 |
| High income | 943 (99.1) | 9 (0.9) | 952 |
| Missing | 1,024 (96.7) | 35 (3.3) | 1,059 |
| **Civil status** |  |  |  |
| Married or in a relationship | 19,797 (97.4) | 535 (2.6) | 20,332 |
| Single or widowed | 6,246 (96.4) | 235 (3.6) | 6,481 |
| Missing | 133 (95.7) | 6 (4.3) | 139 |
| **Smoking** |  |  |  |
| Never | 12,447 (97.9) | 267 (2.1) | 12,714 |
| Previous smoker | 9,444 (96.5) | 347 (3.5) | 9,791 |
| Yes, but not daily | 1,545 (97.9) | 33 (2.1) | 1,578 |
| Yes, daily | 2,428 (95.1) | 124 (4.9) | 2,552 |
| Missing | 312 (98.4) | 5 (1.6) | 317 |
| **Binge drinking** |  |  |  |
| Never | 12,138 (96.2) | 478 (3.8) | 12,616 |
| Less than once a month | 10,085 (97.9) | 219 (2.1) | 10,304 |
| Monthly | 2,663 (98.3) | 47 (1.7) | 2,710 |
| Weekly | 861 (97.5) | 22 (2.5) | 883 |
| Daily or almost daily | 107 (96.4) | 4 (3.6) | 111 |
| Missing | 322 (98.2) | 6 (1.8) | 328 |
| **Depressive symptoms** |  |  |  |
| No | 18,189 (97.5) | 463 (2.5) | 18,652 |
| Yes | 7,870 (96.3) | 306 (3.7) | 8,176 |
| Missing | 117 (94.4) | 7 (5.6) | 124 |

# Table S7. Association between ACEs and type 2 diabetes excluding parental divorce or separation as an ACE (prevalence ratio and 95% CI)

|  | **N (% w/ type 2 diabetes)** | **PR (95% CI)*** |
| --- | --- | --- |
| Total ACE score | 26,952 (2.9) | 1.12 (1.08-1.15) |
| **Number of ACEs** |  |  |
| 0 ACEs | 6,260 (1.9) | 1.00 (ref.) |
| 1 ACE | 6,042 (2.7) | 1.30 (1.03-1.64) |
| 2 ACEs | 4,605 (2.6) | 1.24 (0.96-1.59) |
| 3-4 ACEs | 5,517 (3.3) | 1.56 (1.23-1.96) |
| 5+ ACEs | 4,528 (4.3) | 2.08 (1.64-2.63) |

^*^Adjusted for age and childhood deprivation

# Table S8. Association between ACEs and type 2 diabetes excluding women ever diagnosed with gestational diabetes (prevalence ratio and 95% CI)^*^

|  | **PR (95% CI)**** |
| --- | --- |
| Total ACE score | 1.10 (1.06-1.14) |
| **Number of ACEs** |  |
| 0 ACEs | 1.00 (ref.) |
| 1 ACE | 1.26 (0.96-1.67) |
| 2 ACEs | 1.36 (1.02-1.82) |
| 3-4 ACEs | 1.52 (1.15-2.00) |
| 5+ ACEs | 1.90 (1.43-2.52) |

^*^Women who reported having ever been diagnosed with gestational diabetes (n=1756) were excluded resulting in a final sample of n=25196 women.

^**^Adjusted for age and childhood deprivation

# Table S9. Association between ACEs and type 2 diabetes where women who managed diabetes by a changed diet were classified as not having type 2 diabetes (prevalence ratio and 95% CI)

|  | **PR (95% CI)*** |
| --- | --- |
| Total ACE score | 1.10 (1.06-1.14) |
| **Number of ACEs** |  |
| 0 ACEs | 1.00 (ref.) |
| 1 ACE | 1.31 (0.99-1.74) |
| 2 ACEs | 1.35 (1.01-1.81) |
| 3-4 ACEs | 1.46 (1.10-1.93) |
| 5+ ACEs | 1.96 (1.48-2.59) |

^*^Adjusted for age and childhood deprivation

# Table S10. Association between ACEs and type 2 diabetes in a complete dataset (prevalence ratio and 95% CI)^*^

|  | **PR (95% CI)**** |
| --- | --- |
| Total ACE score | 1.11 (1.08-1.14) |
| **Number of ACEs** |  |
| 0 ACEs | 1.00 (ref.) |
| 1 ACE | 1.32 (1.02-1.70) |
| 2 ACEs | 1.41 (1.08-1.84) |
| 3-4 ACEs | 1.49 (1.15-1.92) |
| 5+ ACEs | 2.06 (1.60-2.65) |

*24,823 women were included in the complete case sample, where women with missing scores on key variables had been excluded

^**^Adjusted for age and childhood deprivation

# Table S11. Association between ACEs and type 2 diabetes stratified by binge drinking frequency and depressive symptoms (prevalence ratio and 95% CI)

|  | **Total ACE score**  **PR (95% CI)*** |
| --- | --- |
| **Binge drinking** |  |
| Never | 1.08 (1.05-1.12)** |
| Less than monthly | 1.15 (1.09-1.22)** |
| Monthly | 1.14 (1.00-1.30) |
| Weekly or more | 1.02 (0.88-1.18) |
| **Depressive symptoms** |  |
| No | 1.09 (1.04-1.14)** |
| Yes | 1.05 (1.00-1.10)** |
| *Adjusted for age and childhood deprivation  **Significant (p<0.05) |  |

# Figure S4. Individual ACE categories adjusted for age, childhood deprivation and BMI


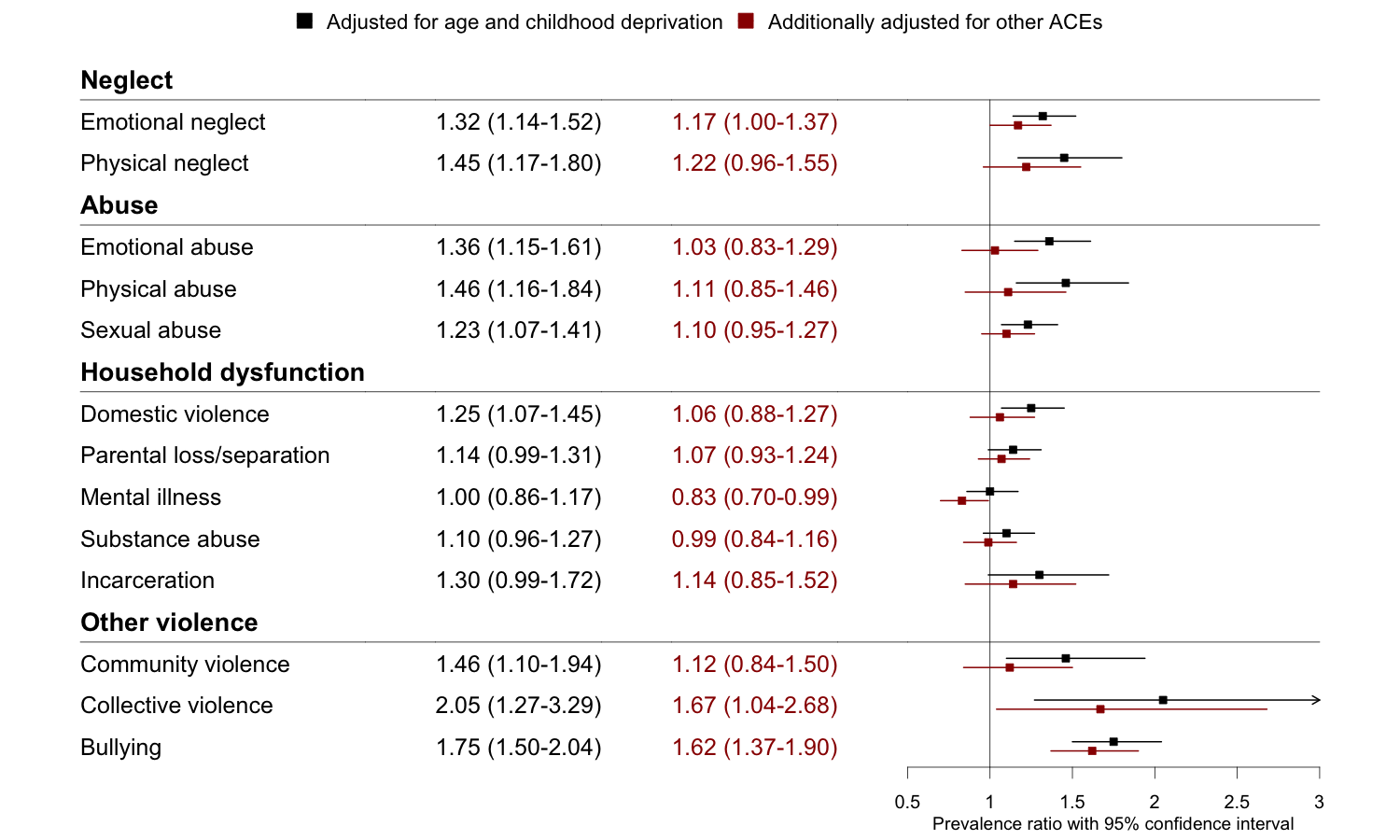


**Table S12. Exposure-mediator associations, (PR and 95% CI)**

| **Exposure** | **Outcome*** | **PR (95% CI)**** |
| --- | --- | --- |
| 5+ ACEs (ref: 0) | BSc education or equivalent | 0.73 (0.68-0.78) |
|  | Medium income | 0.76 (0.67-0.78) |
|  | Married or in a relationship | 0.89 (0.87-0.91) |
|  | BMI under 25 | 0.95 (0.93-0.97) |
|  | Ever smoker | 0.50 (0.48-0.53) |

*Values based on reference groups used in mediation analysis

**Adjusted for age and childhood deprivation

# Figure S5. Subgroup-specific associations between total ACE score and type 2 diabetes, including ACE–covariate interaction terms for each covariate (PR and 95% CI)^*^

^
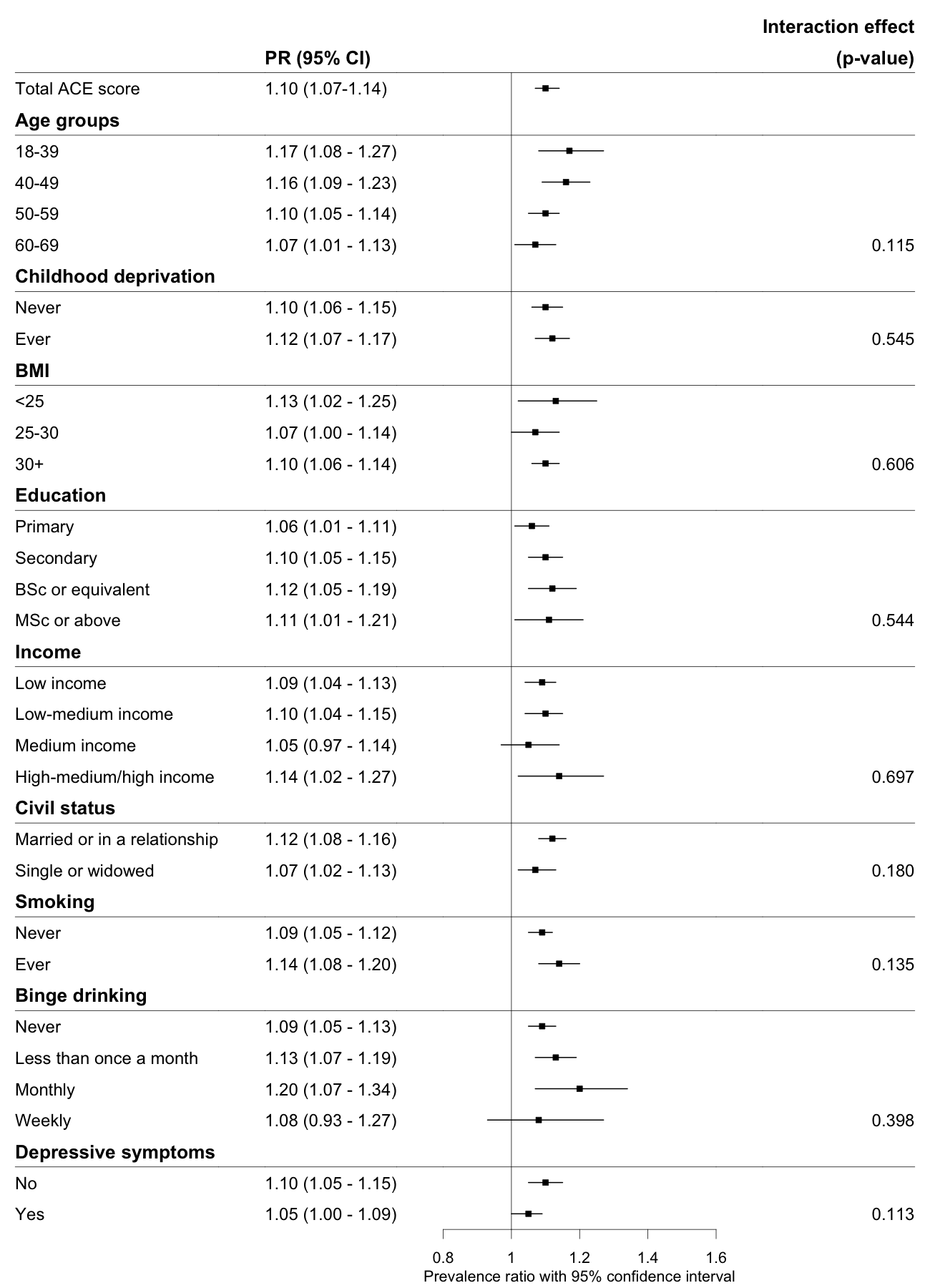
^

^*^Adjusted for age (except when testing ACE-age interaction) and childhood deprivation (except when testing ACE-childhood deprivation interaction)

# Figure S6. Means and standard deviation of age at diagnosis of type 2 diabetes by number of ACEs. Each dot represents one woman with type 2 diabetes in the analytic sample.


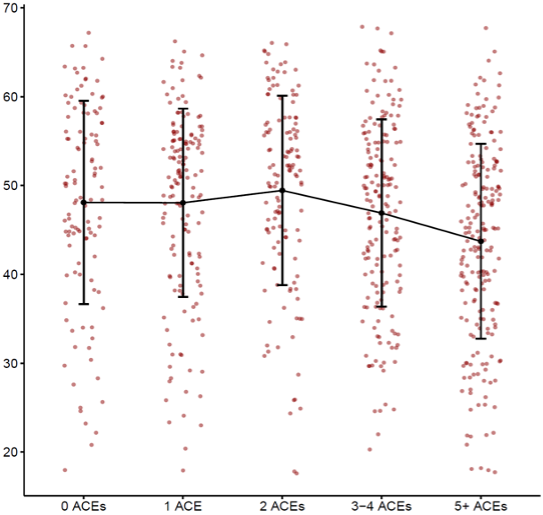

Supplement: ckaf079_Supplementary_Data [file ckaf079_supplementary_data.docx]
